# Construct Validity of the Chinese Version of the Activities of Daily Living Rating Scale III in Patients with Schizophrenia

**DOI:** 10.1371/journal.pone.0130702

**Published:** 2015-06-29

**Authors:** En-Chi Chiu, Yen Lee, Kuan-Yu Lai, Chian-Jue Kuo, Shu-Chun Lee, Ching-Lin Hsieh

**Affiliations:** 1 School of Occupational Therapy, College of Medicine, National Taiwan University, Taipei, Taiwan; 2 Department of Educational Psychology, University of Wisconsin-Madison, Madison, Wisconsin, United States of America; 3 Taipei City Psychiatric Center, Taipei City Hospital, Taipei, Taiwan; 4 Department of Psychiatry, School of Medicine, Taipei Medical University, Taipei, Taiwan; 5 Psychiatric Research Center, Taipei Medical University Hospital, Taipei, Taiwan; 6 Department of Physical Medicine and Rehabilitation, National Taiwan University Hospital, Taipei, Taiwan; Federal University of Rio de Janeiro, BRAZIL

## Abstract

**Background:**

The Chinese version of the Activities of Daily Living Rating Scale III (ADLRS-III), which has 10 domains, is commonly used for assessing activities of daily living (ADL) in patients with schizophrenia. However, construct validity (i.e., unidimensionality) for each domain of the ADLRS-III is unknown, limiting the explanations of the test results.

**Purpose:**

This main purpose of this study was to examine unidimensionality of each domain in the ADLRS-III. We also examined internal consistency and ceiling/floor effects in patients with schizophrenia.

**Methods:**

From occupational therapy records, we obtained 304 self-report data of the ADLRS-III. Confirmatory factor analysis (CFA) was conducted to examine the 10 one-factor structures. If a domain showed an insufficient model fit, exploratory factor analysis (EFA) was performed to investigate the factor structure and choose one factor representing the original construct. Internal consistency was examined using Cronbach’s alpha (α). Ceiling and floor effects were determined by the percentage of patients with the maximum and minimum scores in each domain, respectively.

**Results:**

CFA analyses showed that 4 domains (i.e., leisure, picture recognition, literacy ability, communication tools use) had sufficient model fits. These 4 domains had acceptable internal consistency (α = 0.79-0.87) and no ceiling/floor effects, except the leisure domain which had a ceiling effect. The other 6 domains showed insufficient model fits. The EFA results showed that these 6 domains were two-factor structures.

**Conclusion:**

The results supported unidimensional constructs of the leisure, picture recognition, literacy ability, and communication tool uses domains. The sum scores of these 4 domains can be used to represent their respective domain-specific functions. Regarding the 6 domains with insufficient model fits, we have explained the two factors of each domain and chosen one factor to represent its original construct. Future users may use the items from the chosen factors to assess domain-specific functions in patients with schizophrenia.

## Introduction

Independence in activities of daily living (ADL) is one of the important treatment goals in patients with schizophrenia [1,2]. Patients’ negative symptoms and cognitive impairments (e.g., memory, executive functions, and attention) can influence ADL functions in patients living at home and in the community [3–5]. Patients with schizophrenia who cannot perform ADL independently become a burden to their family members and show poor integration into the community. Assessing ADL functions in patients with schizophrenia is critical for clinicians and researchers in order to develop treatment plans and conduct outcome studies, respectively.

The Activities of Daily Living Rating Scale III (ADLRS-III) is one of the important ADL measures used in Taiwan [6–8] According to the investigation of measures currently used for schizophrenia in psychiatric centers in Taiwan, the ADLRS-II and ADLRS-III are the two commonly used measures to assess ADL function [6]. The ADLRS-III was developed on a basis of the concepts of ADL from several scholars [9–13]. Developers also considered ADL tasks done in current times and users’ experiences of the previous version (the ADLRS-II), and consulted with experts (i.e., occupational therapist, nurse, and social worker) to revise the content (i.e., ensuring expert validity) [14]. The ADLRS-III can be used in the situations of initial and discharge evaluations, as well as progress notes in psychiatric facilities. Previous studies have shown that low ADLRS-III score was associated with low cognition functioning, and severe negative symptoms [7,8]. The ADLRS-III has two advantages: (1) it can be administered in a group setting, which can reduce administrative burden of users; and (2) it can confirm the patient’s knowledge and ability to perform the tasks through patients’ explanations in writing of examples to show their understanding of the items and the abilities to execute the tasks [15].

Some ADL tools have been used in patients with schizophrenia in previous studies, such as the University of California at San Diego Performance-Based Skills Assessment, the Independent Living Skills Survey, and the Functional Remission of General Schizophrenia [16–18]. However, these three measures have no Chinese version. Furthermore, some items/tasks of these measures are culture-specific (e.g., writing a check and using a dishwasher), which are not commonly used in Taiwanese culture. The ADLRS-III was developed considering the cultural appropriateness for patients living in Taiwan. Therefore, at present, the ADLRS-III seems to be the only culturally appropriate ADL measure for patients with schizophrenia in Taiwan.

Construct validity indicates whether a measure can be inferred to measure its underlying constructs. Unidimensionality (a type of construct validity) determines whether the items reflect a single underlying construct [19]. The ADLRS-III is comprised of 10 domains. The sum score of each domain in the ADLRS-III has been used to represent its construct (i.e., domain-specific function) by clinicians and researchers. Validation of unidimensionality of each domain in the ADLRS-III is crucial to ascertain the adequacy of using the sum score of each domain to represent its construct.

In a previous study of patients with mental illness (including schizophrenia and mood disorder), the ADLRS-III has shown acceptable internal consistency (except the personal hygiene domain) and sufficient discriminative validity (except the leisure domain) for distinguishing healthy people and patients with mental illness [14]. However, to our knowledge, no study has examined the unidimensionality of each domain in the ADLRS-III. If unidimensionality is not supported in a domain, the interpretation of the domain with sufficient reliability and discriminative ability is limited and the sum score cannot be used to represent its domain-specific function. Therefore, the main purpose of this study was to examine unidimensionality of each domain in the ADLRS-III. Psychometric properties of a measure are sample dependent and should be validated in the sample (e.g., schizophrenia), which the measure is applied [20]. Thus, we also examined reliability (i.e., internal consistency) and discriminative ability (i.e., ceiling/floor effects) in patients with schizophrenia.

## Methods

### Participants

A retrospective study was conducted and the data were obtained from occupational therapy records at six community rehabilitation centers in northern Taiwan. The ADLRS-III was administered from February 2011 to April 2013 in outpatients with chronic schizophrenia. The eligible participants met the following criteria: diagnosis of schizophrenia based on the Diagnostic and Statistical Manual of Mental Disorders, 4^th^ edition (DSM-IV) and at least an elementary level of education. Patients with schizophrenia who had a history of severe brain injury and diagnoses of mental retardation or substance abuse were excluded. This retrospective study was approved by the Institutional Review Board of Taipei City Hospital in Taipei (TCHIRB-1010811). Patients' records were anonymized and de-identified prior to analysis.

### Procedures

Occupational therapy records were reviewed and eligible participants were selected by an occupational therapist. The self-report data of ADLRS-III and patients’ demographic data were collected from occupational therapy records. The ADLRS-III was administered by six occupational therapists individually in a quiet environment to groups of 10–20 participants at a time. Patients with lower education level could have difficulties in filling out the ADLRS-III (e.g., writing out Chinese characters). In this study, the participants filled out the ADLRS-III without any assistance. If the participants could not write out Chinese characters, they could write out the phonetic symbols instead. If the participants understood the questions, but did not know the answers to the items, they could leave them blank. We scored items according to the answers that they filled out [15].

### Instrument

The ADLRS-III is a self-report questionnaire with 98 items (Appendix in S1 File) related to ADL function in real life. Filling out the ADLRS-III requires 20–45 minutes [15]. The 10 domains of the ADLRS-III are independence, personal hygiene, leisure, picture recognition, current events, literacy ability, money calculation, transportation facilities use, communication tools use, and problem-solving ability. In the independence, personal hygiene, leisure, transportation facilities use, and communication tools use domains, two questions are asked per item. In the first question, patients are required to answer whether they can perform the task or not. In the second question, patients are required to provide examples to confirm that they have the ability to perform the task. The independence domain (rated as 0, 0.5, or 1) assesses the abilities of doing house chores. The personal hygiene domain (rated as 0, 0.5, or 1) assesses the abilities of doing self-cleaning tasks. The leisure domain (rated as 0, 0.5, or 1) assesses the abilities of engaging in leisure activities. The picture recognition domain (rated as 0 or 1) assesses the abilities of identifying the meanings of pictures posted in public places. The current events domain (rated as 0 or 1) assesses the orientation to people, time, and locations. The literacy ability domain (rated as 4 levels [0.5-1-1.5–2] or 2 levels [0–1]) assesses the cognitive abilities of rewriting/correcting characters and recognizing characters on figures. The money calculation domain (rated as 0 or 1) assesses the abilities of mathematical calculation and money usage. The transportation facilities use domain (rated as 3 levels [0–0.5–1] or 2 levels [0–1]) assesses the abilities of taking public transportation, reading signposts, and using a map. The communication tools use domain (rated as 0 or 1) assesses the abilities of using a variety of tools for communication. The problem-solving ability domain (rated as 0, 1, or 2) assesses the abilities of dealing with problems encountered in daily life. The score of each domain ranges from 0–10 points. The leisure domain consists of 14 items. The highest sum score, derived by adding all the points, is 14. However, as stated in the instrument manual, a score of more than 10 will be counted as 10 points. The reason is that not all leisure items are necessary to be administered for examinees in real life. Thus, the developers did not include the scores of four items in the 14-item leisure domain [15].

### Data analysis

The LISREL 9.1 software was used for performing confirmatory factor analysis (CFA). CFA was conducted to evaluate the 10 one-factor structures. The diagonally weighted least squares method was used to estimate CFA parameters [21,22]. We examined goodness-of-fit indices to determine the unidimensional construct of items. Five goodness-of-fit indices were used to examine the level of fit between the overall model and data, such as the ratio of chi-square value to the degrees of freedom, comparative fit index (CFI), Tucker-Lewis index (TLI), and the root mean square error of approximation (RMSEA). The criteria of a good model fit were χ^2^/*df* ≤ 3.0, CFI ≥ 0.95, TLI ≥ 0.95, and RMSEA ≤0.05 [23,24]. RMSEA >0.05 with an upper limit of the 90% confidence interval (CI) ≤0.08 was considered as an acceptable model fit [23,25].

After a domain presented a sufficient model fit, we estimated the factor loadings of the items to represent the correlation between the item and its corresponding factor. The criterion of the factor loading was ≥ 0.40 [26].

If a domain showed an insufficient model fit, it implied that multiple factors may exist in this domain. We conducted exploratory factor analysis (EFA) after CFA to investigate the factor structure of the items, which would be useful for further use and future revision of the domain. For prospective use, we explained factors and chose one factor to represent the original construct [27–29]. The EFA was performed using SPSS 17.0 software. EFA was examined with the principal axis factoring method and the promax oblique rotation. The Kaiser-Meyer-Olkin measure of sampling adequacy (KMO) ≥ 0.80 and the Bartlett test of sphericity with *p* < 0.05 were used to determine whether the data from items of a domain were appropriate to conduct EFA [30,31]. The scree plot and eigenvalues > 1 were used to determine the number of extracted factors [32]. Items with factor loading score ≥ 0.40 were retained [33].

We examined internal consistency and ceiling/floor effects. Internal consistency was examined using Cronbach’s alpha (α). The criterion of Cronbach’s α for group comparison was > 0.70 [34].The ceiling effect was determined by the percentage of participants scoring the maximum score in each domain. The floor effect was determined by the percentage of participants with the minimum score in each domain. A high percentage (≥ 20%) represented ceiling or floor effects [34]. Internal consistency and ceiling/floor effects were also examined for a domain after choosing one factor in the EFA analysis.

## Results

A total of 304 patients with schizophrenia met the recruitment criteria. The mean age of the 304 patients with schizophrenia was 40.0 years, of which 51.5% were female. The average duration of illness was 17.1 years. Around 85% of the participants had an education level of senior high school or above. Further details of the participants are provided in Table 1.

**Table 1:** Characteristics the patients with schizophrenia (n = 304).

Characteristic	
Gender (%)	
Male	48.5
Female	51.5
Age (mean year [SD])	40.0 (10.2)
Onset age (mean year [SD])	22.8 (8.2)
Duration of illness (mean year [SD])	17.1 (9.2)
Education (%)	
Elementary school	3.9
Junior high school	11.3
Senior high school	52.5
College and above	32.3
Schizophrenia subtypes (%)	
Simple type	43.4
Disorganized type	2.3
Paranoid type	18.2
Schizophreniform disorder	1.0
Residual type	1.0
Schizoaffective disorder	5.0
Undifferentiated type	29.1
Level of disability^a^ (%)	
Mild	31.7
Moderate	59.0
Severe	9.3

^a^Level of disability means patients’ degree of mental dysfunction, which was recorded in the disability identification assessed by psychiatrists.

Table 2 shows the results of the four CFA fit indices for the 10 domains of the ADLRS-III. The three domains (i.e., leisure, picture recognition, and literacy ability) met the preset criteria of four fit indices, representing good model fits. The communication tools use domains was considered as an acceptable model fit with χ2/df = 3.0, CFI = 0.98, TLI = 0.97, and an upper limit of 90% CI on RMSEA = 0.08. These 4 domains were further estimated for the factor loadings of their items. The item factor loadings of the 4 domains were >0.40.

**Table 2:** Fit indices of the 10 domains of the ADLRS-III (n = 304).

Domain	χ^2^	*df*	χ^2^/*df*	CFI	TLI	RMSEA (90% CI)
Independence	97.0	35	2.7	0.98	0.97	0.07 (0.05, 0.09)
Personal hygiene	153.8	35	4.3	0.96	0.95	0.06 (0.04, 0.08)
Leisure	146.0	77	1.8	0.98	0.97	0.05 (0.44, 0.07)
Picture recognition	55.2	35	1.5	0.99	0.99	0.02 (0.00, 0.04)
Current events	156.8	35	4.4	0.94	0.92	0.10 (0.09, 0.12)
Literacy ability	42.1	27	1.5	0.99	0.99	0.04 (0.02, 0.07)
Money calculation	401.6	35	11.4	0.68	0.58	0.29 (0.27, 0.31)
Transportation facilities use	192.9	35	5.5	0.96	0.94	0.08 (0.07, 0.10)
Communication tools use	105.8	35	3.0	0.98	0.97	0.06 (0.04, 0.08)
Problem-solving ability	19.8	5	3.9	0.98	0.97	0.09 (0.05, 0.14)

CFI = comparative fit index; TLI = Tucker-Lewis index; RMSEA = root mean square error of approximation; CI = confidence interval.

The other 6 domains did not meet all present criteria for CFA fit indices, showing insufficient model fits. In the EFA analyses, the values of KMO were ***≥*** 0.82 and the Bartlett tests were significant. The 6 domains revealed two-factor structures. Five items with low factor loadings (< 0.40) were deleted (i.e., item 7 and 10 in the independence domain, item 8 in the personal hygiene domain, item 2 in the transportation facilities use domain, and item 7 in the money calculation domain). Table 3 shows the items belonging to the factors and factor loadings (range, 0.43–0.89). We explained the factors and chose a factor to represent the original construct for each domain. In the personal hygiene domain, factor 1 was basic health habit (e.g., “Do you have a habit of washing your hands before eating?”) and factor 2 was neat appearance (e.g., “Do you change into clean clothes before going out?”). Neat appearance was associated with psychotic symptoms in patients with schizophrenia [35]. Thus, we chose “neat appearance” to represent the personal hygiene domain. In the independence domain, factor 1 was house chores done frequently (e.g., “Do you use a mop to clean the floor?”) and factor 2 was house chores done infrequently (e.g., “Do you iron clothing?”). In the current events domain, factor 1 was life events that may change over a short period of time (e.g., “Please name an idol or singer who is currently very popular.”) and factor 2 was life events that may not change over a short period of time. (e.g., “What city do you live in now?”). In the money calculation domain, factor 1 was abilities of mathematical calculation (e.g., “Calculate 18,870 ÷ 34”) and factor 2 was money usage (e.g., “Where can one cash a check?”). In the transportation facilities use domain, factor 1 was usage of transportation (e.g., “Can you take a taxi by yourself?”) and factor 2 was usage of map (e.g., “Please draw the easiest route from Xin-xin market to Chung-Hsing middle school on the map.”). In the problem-solving ability domain, factor 1 was ability to handle external issues (e.g., “Please provide three ways to deal with someone who is bullying you.”) and factor 2 was ability to deal with self-emotion (e.g., “Please provide three ways to deal with a bad mood.”). In the 5 domains (i.e., independence, current events, money calculation, transportation facilities use, and problem-solving ability), their factor 1 was chosen, because the concepts of factor 1 were closer to the original construct.

**Table 3:** Factor loadings of the 6 domains with two-factor structure using exploratory factor analysis.

Domain	Factor	Item	Factor loading
Independence	1. Doing house chores frequently	1. Mopping floor	0.43
		2. Doing laundry	0.50
		4. Washing dishes	0.43
		8. Shopping for daily supplies	0.75
		9. Shopping for clothes or shoes	0.80
	2. Doing house chores rarely	3. Ironing clothes	0.76
		5. Sewing on a button	0.69
		6. Cooking	0.58
Personal hygiene	1. Basic health habit	1. Brushing teeth	0.63
		2. Washing face	0.70
		3. Washing hands	0.66
		10. Wearing shoes	0.68
	2. Neat appearance	4. Washing hair	0.50
		5. Combing hair	0.46
		6. Taking a shower	0.71
		7. Changing underwear	0.89
		9. Changing clean clothes	0.54
Current events	1. Life events may change over a short period of time	2. Naming idol	0.53
		3. Naming physician	0.66
		4. Naming political party	0.67
		5. Naming number of working days	0.57
		6. Naming national headline event	0.58
		8. Naming tourist attraction	0.64
		10. Naming fast food restaurant	0.69
	2. Life events may not change over a short period of time	1. Naming current president	0.84
		7. Naming city of residence	0.69
		9. Naming convenience store	0.72
Money calculation	1. Abilities of mathematical calculation	1. Solving equation 1	0.71
		2. Solving equation 2	0.71
		3. Solving calculation question 1	0.72
		4. Solving calculation question 2	0.76
		5. Solving calculation question 3	0.76
	2. Money usage	6. Naming largest value of paper bill	0.74
		8. Cashing check	0.72
		9. Paying utility bill	0.74
		10. Estimating monthly expenses	0.61
Transportation facilities use	1. Usage of transportation	1. Riding bicycle	0.49
		3. Taking taxi	0.58
		4. Taking bus	0.54
		5. Naming bus company	0.65
		6. Naming train	0.65
		7. Reading signpost 1	0.70
		8. Reading signpost 2	0.64
	2. Usage of map	9. Drawing route	0.83
		10. Writing names of roads	0.84
Problem-solving ability	1. Handling external issues	1. Finding new address	0.64
		3. Dealing with bully	0.70
		4. Handling friend’s problems	0.73
		5. Handling bad family relations	0.72
	2. Handling self-emotion	2. Handling bad mood	0.84

Cronbach’s α of the 10 domains were 0.79–0.87 (Table 4). We observed ceiling effects in the personal hygiene, leisure, and current events domains (22.7%-52.6%). No floor effects were found in the 10 domains (1.3%-10.9%). Cronbach’s α of the 6 domains after choosing factors in the EFA analyses (0.73–0.85) were lower than those of the original version, except the money calculation domain. For the chosen factors of the 6 domains, we found ceiling effects (27.3%-53.3%), except the problem-solving ability, and floor effect in the money calculation domain (23.7%).

**Table 4:** Descriptive statistics and Cronbach’s α of the 10 domains of the ADLRS-III (n = 304).

Domain	Number of items		Internal consistency (α)		Ceiling effect (%)		Floor effect (%)	
	Original version	Chosen factor^a^	Original version	Chosen factor^a^	Original version	Chosen factor^a^	Original version	Chosen factor^a^
Independence	10	5	0.80	0.74	19.8	53.3	1.3	1.0
Personal hygiene	10	5	0.80	0.73	22.7	52.0	2.0	2.6
Leisure	14	-	0.79	-	23.7	-	2.3	-
Picture recognition	10	-	0.87	-	17.1	-	10.2	-
Current events	10	7	0.85	0.81	52.6	52.6	1.6	3.3
Literacy ability	9	-	0.81	-	4.3	-	10.9	-
Money calculation	10	5	0.84	0.85	6.3	27.3	6.6	23.7
Transportation facilities use	10	7	0.83	0.80	13.2	31.3	5.3	5.3
Communication tools use	10	-	0.80	-	11.6	-	2.3	-
Problem-solving ability	5	4	0.84	0.82	16.8	17.8	6.9	10.2

^a^After conducting the EFA analysis, one factor was chosen to represent its original construct. Symbol “-”indicates no conducting the EFA analysis.

## Discussion

This study used CFA to determine the factor structure of the ADLRS-III in patients with schizophrenia. One-factor structure analysis of the 4 domains (i.e., leisure, picture recognition, literacy ability, and communication tools use) presented sufficient model fits. The results indicate that the construct validity of these 4 domains was supported for measuring its unidimensional construct. Thus, the items’ scores of the individual domains can be summed up to represent patients’ level of functioning. Moreover, the item factor loadings of these 4 domains were sufficient, indicating that each item was an acceptable indicator of the domain which it belongs to [36]. In clinical implications, clinicians can use the sum scores of these 4 individual domains of the ADLRS-III to identify patients’ domain-specific functions, develop treatment plans, and follow-up on patients’ ADL functions in the initial evaluation and re-evaluation of patient progress. Clinicians can also use the sum scores of these domains to communicate and cooperate with other healthcare professionals in a multidisciplinary approach.

The 4 domains with sufficient model fits were not found to have ceiling and floor effects, except for the leisure domain which had a ceiling effect. That is, the 3 domains (i.e., picture recognition, literacy ability, and communication tools use domains) are about to discriminate the participants’ domain-specific function within the high and low score ranges. The leisure domain distinguished participants with low leisure ability, but not those with high leisure ability. One possible reason for the ceiling effect is the method of calculating the sum score. The leisure domain contains 14 items, but the sum score (> 10) is counted as 10 points. If the original sum score is used (e.g., the sum score 11 is counted as 11 points), no notable ceiling effect (2.0%) was found. Thus, we recommend using the original sum score to decrease the ceiling effect in the leisure domain. These 4 domains demonstrated acceptable internal consistency, indicating the interrelatedness of the items. Thus, the patients could have consistent answers to the items within each domain. Overall, the absence of notable ceiling and/or floor effects and the sufficient internal consistency further support the psychometric properties of these 4 domains.

The other 6 domains of the ADLRS-III in the CFA analyses showed insufficient model fits, such as independence, personal hygiene, current events, money calculation, transportation facilities use, and problem-solving ability. That is, the items of each domain do not measure a unidimensional construct. Moreover, the results of the EFA analyses showed two-factor structures. Thus, the sum scores of these 6 individual domains could not be used to represent its domain-specific function in patients with schizophrenia. In this study, we chose one factor to represent the original construct. Clinicians and researchers may use the sum scores of the items from the chosen factors to assess domain-specific functions. However, the chosen factors with less number of items might have insufficient psychometric properties. For example, we observed decreasing Cronbach’s α (5 items [0.73] vs. 10 items [0.80]) in the personal hygiene domain, and obvious ceiling effect (27.3%) and floor effect (23.7%) in the 5-item money calculation domain. Comparing the ADLRS-III (i.e., self-report) to other clinician-reported and family/caregiver reported measures, the self-report measure requires relatively low cost, little staff time, less concerns to identify which family/caregiver is able to report (especially for patients who are middle aged and older), and no highly trained raters. However, the reliability of patients’ self-reports may be influenced by lack of insight, personal values, and situational events [37,38].Therefore, future users may consider using additional measures or other modes of administration (e.g., clinician-reported or family/caregiver reported) to supplement these domains. Moreover, future studies may revise these 6 domains of the ADLRS-III according to our findings.

Although the ADLRS-III has not been used in studies of Western countries, it is able to be applied in the Western population for three reasons. First, the ADLRS-III was developed based on the concepts of ADL from Western countries [9–13]. Second, most of the ADL items of the ADLRS-III are regularly performed in the Western population (e.g., doing laundry, changing clean clothes, and watching TV). Third, the ADLRS-III provides 10 domains to understand patients’ ADL functions in a comprehensive manner. Clinicians and researchers in other countries may adjust parts of items of the ADLRS-III according to cultural considerations.

This study has 3 limitations. First, our data were collected from occupational therapy records (secondary data) and the quality of data may be unstable. Further studies are warranted to cross-validate our results. Second, we did not find the evaluation records of the cognitive level and the psychiatric condition of participants from occupational therapy records, which may limit our knowledge on what kind of patients are suitable for the ADLRS-III. Third, the data was collected from six community rehabilitation centers located in northern Taiwan. Our findings may limit the generalization to other places, especially the Western population.

In summary, unidimensionality was supported in the 5 domains (i.e., independence, leisure, picture recognition, literacy ability, and communication tools use) of the ADLRS-III. The sum scores of these 5 domains were appropriate for representing their domain-specific functions. Moreover, these 5 domains had acceptable internal consistency and no ceiling/floor effects, except the leisure domain with a ceiling effect. The other 5 domains with insufficient model fits showed two-factor structures in the EFA analyses. We have chosen one factor for each domain to represent its original construct for future use.

## Supporting Information

S1 File Appendix. Item description of the 10 domains of the ADLRS-III.
(PDF)
