# Supplementary material for: Construct Validity of the Chinese Version of the Activities of Daily Living Rating Scale III in Patients with Schizophrenia
Source: PLoS One. 2015 Jun 29;10(6):e0130702. doi: 10.1371/journal.pone.0130702 (PMC4488143; doi:10.1371/journal.pone.0130702)
Supplement: S1 File — Item description of the 10 domains of the ADLRS-III. (PDF) [file pone.0130702.s001.pdf]

Domain	Item
Independence	1. Do you use a mop to clean the floor? How many times do you need to mop the floor for it to be clean?
	2. Do you do the laundry yourself? What type of detergent have you used in the past?
	3. Do you iron clothing? At what age did you learn how to iron clothes?
	4. Do you wash the dishes? What type of dishwashing liquid have you used in the past?
	5. Do you know how to use needle and thread to sew on a button? At what age did you learn how to sew on a button?
	6. Do you cook? What dishes have you cooked in the past?
	7. Do you go grocery shopping yourself? Where did you buy the groceries?
	8. Do you go shopping for daily supplies? What daily supplies have you bought in the past?
	9. Do you shop for clothes or shoes yourself? Where did you shop?
	10. Do you repair broken items? What items have you fixed in the past?
Personal hygiene	1. Do you have a habit of brushing your teeth in the morning and evening? How many times do you brush your teeth each day?
	2. Do you have a habit of washing your face after waking up? How many times do you wash your face each day?
	3. Do you have a habit of washing your hands before eating? Do you have a habit of washing your hands after going to the restroom?
	4. Is your hair oily, normal, or dry? How often do you wash your hair?
	5. What hair style do you have now? How many times do you comb your hair each day?
	6. At the moment, do you need people to remind you to take a shower? How often do you take a shower?
	7. Do you change your underwear after taking a shower? How often do you change your underwear?
	8. Are you in the habit of using cosmetics or skincare products? What products have you used in the past?
	9. Do you change into clean clothes before going out? How often do you change your clothes?
	10. Do you wear shoes (socks) when going out? Are you in the habit of washing your feet every day?
Leisure	1. Do you have a habit of watching TV? Please name the type of TV show you watch most often.

	2. Do you play chess? Please name the type of chess you play.
	3. Do you play mahjong? How many tiles do you play in a set?
	4. Do you play video games? Please name the type of video game you play.
	5. Do you exercise? Please name the type of exercise you do.
	6. Do you go hiking in the mountains? Please name a mountain where you have been hiking.
	7. Do you go fishing? Please name a place where you have been fishing.
	8. Are you in the habit of going shopping? Please name a place where you shop most often.
	9. Do you do photography? Please name a type of camera you have used in the past.
	10. Do you do arts and crafts? Please name the activity you do most often
	11. Do you sing or go to karaoke? Please name a song you can sing.
	12. Do you play musical instruments? Please name an instrument you can play.
	13. Do you collect items? Please name a type of item you have collected in the past.
	14. Do you do gardening? Please name a type of plant you have planted in the past.
Picture recognition	1. What is the meaning of the picture? (for people with disability)
	2. What is the meaning of the picture? (escalator)
	3. What is the meaning of the picture? (restroom for men or women)
	4. What is the meaning of the picture? (traffic light for pedestrians)
	5. What is the meaning of the picture? (fire extinguisher)
	6. What is the meaning of the picture? (priority seat)
	7. What is the meaning of the picture? (highway number)
	8. What is the meaning of the picture? (kitchenette)
	9. What is the meaning of the picture? (bowling alley)
	10. What is the meaning of the picture? (drinking is prohibited)
Current events	1. Who is your country's current president?
	2. Please name an idol or singer who is currently very popular.
	3. Please name a physician that you are familiar with.
	4. Which political party currently has the majority of seats in your country?
	5. How many days does a government officer currently have to work each week?

	6. Please name a recent national headline event.
	7. What city do you live in now?
	8. Please name a tourist attraction with good scenery.
	9. Please name a convenience store.
	10. Please name a fast food restaurant.
Literacy ability	1. Circle the wrong characters and correct them.
	2. Write these English words in lower case (i.e., DAILY FUNCTION).
	3. Write these numbers in Chinese upper-case for a withdrawal slip (i.e., 25964)
	4. Change these Roman numerals into numbers (i.e., IV, VII, II, IX)
	5. What is the meaning of the picture? (28.5℃)
	6. What is the meaning of the picture? (EXIT)
	7. What is the meaning of the picture (EMERGENCY)
	8. What is the meaning of the picture? (FAX)
	9. What is the meaning of the picture? (VISA)
Money calculation	1. Calculate 18,870 ÷ 34
	2. Calculate 893 × 57 + 1099
	3. A lunch box costs $89. If you pay $500 for 4 lunch boxes, how much change will you get?
	4. An item of clothing costs $6300. How much do you pay if it is 25% off?
	5. If you buy 2 cans of soda ($18 each) and 8 loaves of bread ($15 each), how much money will you spend in total?
	6. What is currently the largest value of paper bill in your country?
	7. Do you have a credit card? Which bank is it from?
	8. Where can one cash a check?
	9. Where can you pay a utility bill?
	10. How much are your monthly expenses?
Transportation facilities use	1. Can you ride a bicycle? At what age did you learn how to ride a bicycle?
	2. What kind of driver’s license do you have? At what age did you get your driver’s license?
	3. Can you take a taxi by yourself? How much is the initial fare for a taxi ride?
	4. Can you take a bus by yourself? Which bus route do you take most often?
	5. Which bus company can you take to travel from Taipei to Kaohsiung?

	6. Which type of train travels the fastest in your country?
	7. Please look at the signpost in the picture on the right. If you head straight, what is the name of the road?
	8. Please look at the signpost in the picture on the right. If you head right, what is the name of the road?
	9. Please study the map below. Please draw the easiest route from Xin-xin market to Chung-Hsing middle school on the map.
	10. Please study the map below. Please write down the names of the roads you took from Xin-xin market to Chung-Hsing middle school.
Communication tools use	1. Aside from using coins, how else can you use a public phone?
	2. What is the emergency telephone number for contacting the police?
	3. What is the current first 4-digit number for making toll-free calls?
	4. How much is the stamp for a regular mail?
	5. What is the color of the mailbox for express post?
	6. Where do you have to go to send a package?
	7. Do you have a cell phone? How much is the average monthly bill?
	8. Do you know how to use a computer? What type of Chinese input method do you use?
	9. Do you have an e-mail address? Please write down your e-mail address.
	10. Please write down the web address of a website homepage.
Problem-solving ability	1. Please provide three ways of finding a new address.
	2. Please provide three ways to deal with a bad mood.
	3. Please provide three ways to deal with someone who is bullying you.
	4. Please provide three ways to deal with your friend who is asking for help.
	5. Please provide three ways to deal with bad family relations.
